# Screening costs associated with donor selection for fecal microbiota transplantation for treatment of PD-1 refractory melanoma patients

**DOI:** 10.1097/CMR.0000000000000871

**Published:** 2023-02-17

**Authors:** Dylan Fortman, Maria G. Pazan Avellan, Drew Hurd, Marc Schwartz, Howard Dubner, Corey Hewitt, Samantha Berton, Scarlett Ernst, Amy Rose, Hong Wangd, Hassane Zarour, Diwakar Davar

**Affiliations:** a Division of Internal Medicine, Department of Medicine; b Department of Medicine, UPMC Hillman Cancer Center; c Division of Gastroenterology, Department of Medicine; d Department of Biostatistics; e Department of Immunology and fDivision of Hematology-Oncology, Department of Medicine, University of Pittsburgh, Pittsburgh, Pennsylvania, USA

## Abstract

The gut microbiome acts as a tumor-extrinsic regulator of responses to immune-checkpoint inhibitors (ICIs) targeting PD-1 and CTLA-4 receptors. Primary resistance to anti-PD-1 ICI can be reversed via responder-derived fecal microbiota transplant (FMT) in patients with refractory melanoma. Efforts to create stool banks for FMT have proved difficult. Therefore, we aimed to establish a novel donor-screening program to generate responder-derived FMT for use in PD-1 refractory melanoma. Candidate PD-1 responder donors and PD-1 refractory recipients were recruited via clinic-based encounters at the University of Pittsburgh Medical Center hospitals. Eligible donors and recipients underwent physician assessment and screening of serum, stool and nasopharynx for transmissible agents, which included SARS-CoV-2 modification. The cost of donor and recipient screening was calculated. Initially, 29 donors were screened with 14 eligible donors identified after exclusion; of the 14 donors, eight were utilized in clinical trials. The overall efficiency of screening was 48%. Seroprevalence rates for cytomegalovirus, Epstein-Barr virus, HSV-2, HHV-6, HTLV-1, HTLV-2, and syphilis were similar to published statistics from healthy blood donors in the USA. Donor stool studies indicated a 3.6% incidence of E. histolytica and norovirus, 3.7% incidence of giardia and 7.1% incidence of C. difficile. A single donor tested positive for SARS-CoV-2 in stool only. The cost for finding a single eligible donor was $2260.24 (pre-COVID) and $2,460.24 (post-COVID). The observed screening efficiency suggests that a well-resourced screening program can generate sufficient responder-derived donor material for clinical trial purposes. Eliminating testing for low-prevalence organisms may improve cost-effectiveness.

## Introduction

Fecal microbiota transplant (FMT) hasbeen commonlyused to treat recurrent *Clostridium difficile* infections (rCDI), a hospital-acquired infectiontypically treated with prolonged courses of antibiotics, which maintain and exacerbate intestinal dysbiosis [1]. FMT restores normal gut ecology, and in this context is well-tolerated and efficacious with cure rates of 85% in rCDI [2–5].

Cancer immunotherapy, particularly immune-checkpoint inhibitors (ICI) targeting cytotoxic T-lymphocyte-associated antigen 4 (CTLA-4) and programmed death 1 (PD-1) inhibitory immune checkpoints, has transformed the management of advanced cancers. In addition to tumor-intrinsic mechanisms mediating response and resistance to ICI, the role of the gut microbiome as a tumor-extrinsic regulator of responses to anti-PD-1 [6–10], and anti-CTLA-4 [11,12], is increasingly appreciated. Although multiple studies have reported that a favorable gut microbiome is associated with response to anti-PD-1 in cancer patients, its precise composition is not yet fully understood [13–15], and the concordance among identified species in different studies is limited [13–17].

Given the role of gut microbiota in mediating nonresponse in ICI-treated melanoma, several clinical trials have studied the role of microbiome interventions in treating PD-1 nonresponsive cancers with interventions ranging from probiotics, single strain bacterial isolates (MRx0518 and EDP1503), defined bacterial consortia (VE800 and SER301) and FMT [18]. Of these, given the as-yet-undefined significant complexity underlying immune-tumor-microbiome interactions, FMT represents the most direct method of microbiome modulation by which beneficial bacteria can be introduced into an established complex ecosystem along with other microorganisms that may play supportive roles.

To evaluate the role of microbiome modulation in reversing primary resistance to anti-PD-1 ICI, we previously conducted a feasibility trial to evaluate the safety and efficacy of responder-derived FMT (R-FMT) together with anti-PD-1 pembrolizumab in PD-1 refractory metastatic melanoma patients [19]. In this study, we demonstrated that single administration of R-FMT resensitized patients with primary refractory melanoma to pembrolizumab: with objective responses in three patients and durable stable disease (SD) lasting >12 months in three patients (i.e. objective response rate 20% and disease control rate 40%) with a low rate of toxicity [19]. Response to R-FMT was associated with enrichment of taxa belonging to phyla Firmicutes and Actinobacteria in post-FMT fecal samples in recipients, along with reduced CXCL8/IL-8 expressing myeloid cells intratumorally, and increased CD56+CD8+ T cells, CD45RA/CD4 positive effector memory T cells and mucosal-associated invariant T cells *peripherally* [19]. Similar results were observed in another trial of R-FMT in PD-1 refractory melanoma [20].

These results have spurred the efforts to create local stool banks containing ready-to-use, high-quality, donor fecal material for use in immuno-oncologic studies such as the ones described above. Given concerns regarding the potential transmission of pathogenic organisms and other noninfectious traits putatively mediated by gut microbiome (metabolic syndrome and fibromyalgia, etc.); candidate stool donors and donor fecal material typically undergo extensive pretransplant screening and testing [21]. In the rCDI context, a recent study that evaluated the stool of candidate FMT donors to determine fitness for clinical use reported that only 3% of donors passed the quality control assessment [22]. Herein, we report on our experience in establishing a novel donor-screening program to generate R-FMT for use in PD-1 refractory melanoma. We report on the success rate of history and serologic screening, and seroprevalence of latent viral, bacterial and parasitic organisms in this unique cohort. We compare the donor-screening methodology used herein with other programs that have published screening methods and acceptance rates albeit in nononcology indications [23–27]. Finally, we review the cost and feasibility of establishing such a screening program.

## Methods

### PD-1 responder donor recruitment

Candidate PD-1 responder donors were recruited through clinic-based encounters at the Hillman Cancer Center in Pittsburgh, Pennsylvania which is affiliated with the University of Pittsburgh Medical Center (UPMC) hospital system, including UPMC Shadyside and UPMC Presbyterian Hospitals. Recruitment of donors was divided into two phases: pre-SARS-coronavirus-2 (SARS-CoV02) infection (COVID) and post-COVID. The pre-COVID phase of recruitment took place from May 2018 to December 2019; the post-COVID phase started in March 2021 and concluded in July 2022. Candidate PD-1 responder donors were identified based on a documented history of metastatic cutaneous (or unknown primary) melanoma treated with nivolumab, pembrolizumab or other approved/investigational anti-PD-(L)1 ICI administered singly. Enrollment was restricted to PD-1 responder patients who were currently in durable remission, defined as median duration of remission lasting ≥12 months (for complete responders) or ≥24 months (for partial responders) measured since initiation of therapy, as published data indicates that the likelihood of relapse was negligible in ICI-treated melanoma patients with the durable response at this duration of follow-up [28–30]. Other eligibility criteria included: absence of recent antibiotic treatment (defined as oral/parenteral within 1 month of donation); any history of significant gastrointestinal illnesses, including active inflammatory bowel disease, active irritable bowel syndrome, any chronic diarrheal disorder, active primary gastrointestinal malignancy or major gastrointestinal surgical procedures; history of symptomatic autoimmune illness; history of documented chronic pain syndromes (fibromyalgia or chronic fatigue); history of severe (35–39.9 kg/m^2^) or morbid (>40.0 kg/m^2^) obesity based on BMI; and active pregnancy. Patients with suspected SARS-CoV-2/COVID infection diagnosed per Center for Disease Control and Prevention (CDC) guidelines were excluded in the post-COVID phase of the study.

**Fig. 1:**
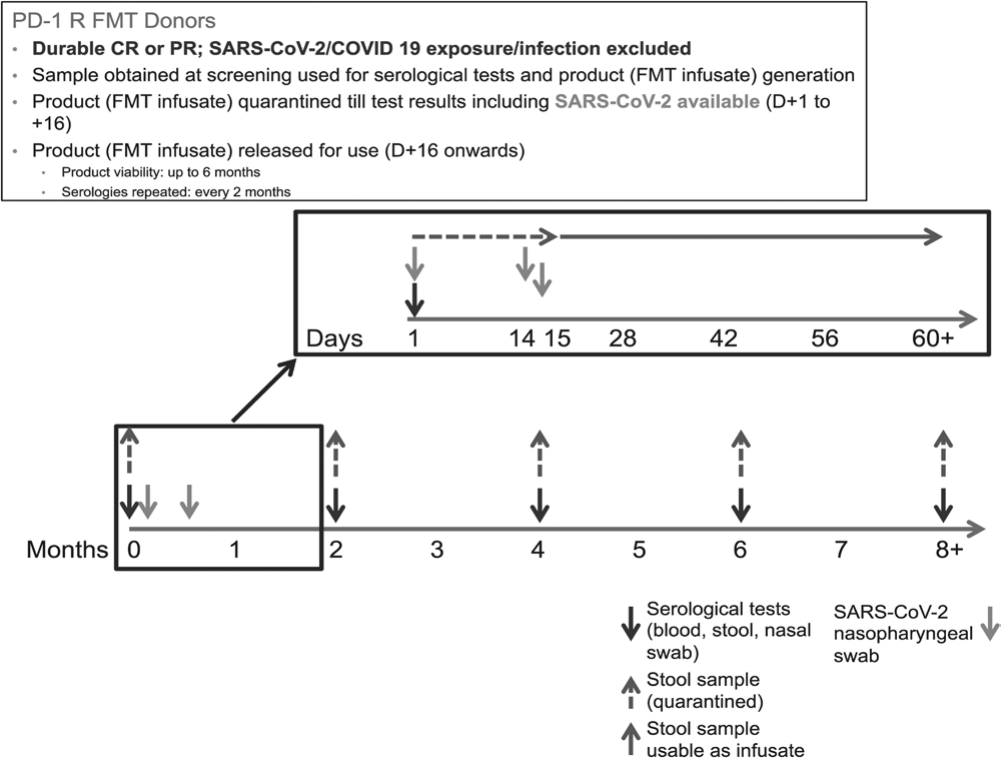
Donor and recipient screening protocol.

Donor health was continuously monitored throughout their donation lifetime. FMT studies in non-oncological indications have typically utilized bookend testing, wherein blood and stool tests are performed twice on either end of a collection period that typically spans 60 days. However, given the novel nature of this study (PD-1 refractory melanoma), the use of a previously uncharacterized source of FMT (PD-1 durable responders) and the uncertain performance of bookend testing in these circumstances, all tests were repeated every 14 days during the donor testing phase as illustrated in Fig. 1 **(Fig. 1: Donor and Recipient Screening Protocol).** Samples were quarantined until the donor passed screening at the end of the collection window.

### PD-1 responder donor screening

Candidate PD-1 responder donors who expressed a willingness to participate were screened for exposures to infectious agents using a previously published methodology for the use of FMT to treat rCDI,^31^ that consisted of a specific questionnaire (Supplementary Table 1, Supplemental digital content 1, http://links.lww.com/MR/A307). Candidate donors who declined participation were asked no further questions. If candidate PD-1 responder donors passed initial screening criteria, they were asked to provide written informed consent, received a full history and physical examination by a physician and underwent stool, blood and nasopharyngeal testing for potentially transmissible infectious agents serologic and fecal testing of infectious agents listed in Table 1.

**Table 1:** Infectious screening assays on blood, stool and nasopharyngeal specimens

Infectious agent	Assay	Nature of sample (blood, stool other)	Lab processing test	Assay manufacturer	Interpretation of result
Cytomegalovirus (CMV)	CMV IgG	Blood	PUH ID Lab	Bio-Rad immunoassay (ToRc IgG)	Seromatching required between donors/recipients.
Epstein-Barr virus (EBV)	EBV IgG and IgM	Blood	PUH ID Lab	Bio-Rad immunoassay (EBV IgG and IgM)	Seromatching required between donors/recipients.
Entameba histolytica	ELISA	Blood	Quest Diagnostics via SHY ID Lab	Quest	• Excluded from donating if positive.
					• Referral to infectious diseases if infected.
Hepatitis A virus (HAV)	HAV total antibody (anti-HAV IgM and IgG)	Blood	Quest diagnostics via SHY ID Lab	Quest immunoassay	• Excluded from donating if positive.
					• Referral to infectious diseases.
Hepatitis B virus (HBV)	HBV surface antigen (HBsAg), HBV surface antibody (anti-HBs), and HBV core antibody (anti-HBc)	Blood	PUH ID Lab	Siemens immunoassay (HBsAg, HBc Total, anti-HBs2)	• Only immune and non-immune/uninfected individuals permitted to donate.
					• Referral to infectious diseases if infected.
Hepatitis C virus (HCV)	Hepatitis C antibody (anti-HCV) with reflex HCV RNA	Blood	PUH ID Lab	Siemens immunoassay (HCV) with reflex confirmation using Quest	• Excluded from donating if positive (anti-HCV reactive, and HCV RNA positive)
					• Referral to infectious diseases
Herpes simplex virus (HSV) type 1 (HSV-1)	HSV-1 IgG	Blood	PUH ID Lab	Bio-Rad immunoassay (HSV-1/2 IgG)	Seromatching required between donors/recipients.
Herpes simplex virus (HSV) type 2 (HSV-2)	HSV-2 IgG	Blood	PUH ID Lab	Bio-Rad immunoassay (HSV-1/2 IgG)	Seromatching required between donors/recipients.
Human herpesvirus 6 (HHV-6)	HHV-6 IgG	Blood	Quest diagnostics via SHY ID Lab	Quest immuno-fluorescence assay	Seromatching required between donors/recipients.
Human polyomavirus-2 (JC virus)	JC virus RT PCR	Blood	Quest diagnostics via SHY ID Lab	Quest RT-PCR	Seromatching required between donors/recipients.
Human T-lymphotropic virus (HTLV)-1	HTLV-1 IgG	Blood	Quest diagnostics via SHY ID Lab	Screen immunoassay, confirmatory immunoblot Quest	• Excluded from donating if positive.
					• Referral to infectious diseases.
Human T-lymphotropic virus (HTLV)-2	HTLV-2 IgG	Blood	Quest diagnostics via SHY ID Lab	Screen immunoassay, confirmatory immunoblot Quest	• Excluded from donating if positive.
					• Referral to infectious diseases.
Human immunodeficiency virus (HIV)	5th generation IgG with reflex ELISA	Blood	PUH ID Lab	Bio-Rad immunoassay (HIV Ag-Ab)	• Excluded from donating if positive.
					• Referral to infectious diseases.
Strongyloides stercoralis	S. stercoralis IgG	Blood	Quest diagnostics via SHY ID Lab	Quest immunoassay	• Excluded from donating if positive.
					• Referral to Infectious Diseases.
Syphilis	RPR, IgM, IgG	Blood	PUH ID Lab	T pallidum antibody immunoassay reflex RPR screen Bio-Rad	• Excluded from donating if positive.
					• Referral to infectious diseases.
Campylobacter species	Culture	Stool	PUH ID Lab	Diasorin, Verigene	• Excluded from donating if positive.
Clostridium difficile (C. diff)	C. diff toxin EIA with reflex PCR	Stool	PUH ID Lab	EIA (Alere), PCR (Cepheid)	• Excluded from donating if positive.
Helicobacter pylori (H. pylori)	EIA	Stool	Quest diagnostics via SHY ID Lab	Quest	• Excluded from donating if positive.
Salmonella	Culture	Stool	PUH ID Lab	Diasorin, Verigene	• Excluded from donating if positive.
Shigella	Culture	Stool	PUH ID Lab	Diasorin, Verigene	• Excluded from donating if positive.
Vibrio	Culture	Stool	PUH ID Lab	Diasorin, Verigene	• Excluded from donating if positive.
Yersinia	Culture	Stool	PUH ID Lab	Diasorin, Verigene	• Excluded from donating if positive.
Rotavirus	Culture	Stool	PUH ID Lab	Diasorin, Verigene	• Excluded from donating if positive.
					• May be reconsidered for donation after 12 weeks from the initial positive test, if the subsequent test is negative.
Adenovirus	Adenovirus DNA RT PCR	Stool	Quest diagnostics via SHY ID Lab	Quest	• Excluded from donating if positive.
					• May be reconsidered for donation after 12 weeks from the initial positive test, if the subsequent test is negative.
Norovirus	Culture	Stool	PUH ID Lab	Diasorin, Verigene	• Excluded from donating if positive.
					• May be reconsidered for donation after 12 weeks from the initial positive test, if the subsequent test is negative.
Giardia	EIA	Stool	PUH ID Lab	Quik Chek, Alere	• Excluded from donating if positive.
					• May be reconsidered for donation after 12 weeks from the initial positive test, if the subsequent test is negative.
Cryptosporidia	EIA	Stool	PUH ID Lab	Quik Chek, Alere	• Excluded from donating if positive.
					• May be reconsidered for donation after 12 weeks from the initial positive test, if the subsequent test is negative.
Enterovirus	RT-PCR	Stool	Quest diagnostics via SHY ID Lab	Quest	• Excluded from donating if positive.
Enterohemorrhagic E. Coli (EHEC)	NAAT	Stool	Quest Diagnostics via SHY ID Lab	Quest	• Excluded from donating if positive.
Vancomycin-resistant Enterococcus (VRE)	Culture	Stool	PUH ID Lab	SpectraVRE chromagar, Remel	• Excluded from donating if positive.
Carbapenem-resistant Enterobacteriaceae (CRE)	Culture	Stool	PUH ID Lab	CRE VRE chromagar, Hardy Diagnostics	• Excluded from donating if positive.
Extended-spectrum β-lactamase-producing Enterobacteriaceae (ESBL)	Culture	Stool	PUH ID Lab	ESBL chromagar, Hardy Diagnostics	• Excluded from donating if positive.
SARS-CoV-2	COVID-19 rtPCR	Stool	DSL Laboratories	DSL Laboratories	• Excluded from donating if positive
					• Prohibited from donating for 12 weeks following this exposure.
					• Before being reconsidered for donations, patients must undergo bookend testing as outlined and test negative.
SARS-CoV-2	COVID-19 rtPCR	Nasopharyngeal swab	DSL Laboratories	DSL Laboratories	• Excluded from donating if positive
					• Prohibited from donating for 12 weeks following this exposure.
					• Before being reconsidered for donations, patients must undergo bookend testing as outlined and test negative.
Methicillin-resistant Staphylococcus aureus (MRSA)	MRSA culture	Nasopharyngeal swab	Quest Diagnostics via SHY ID Lab	MRSA chromagar	• Excluded from donating if positive.

Presbyterian Infectious Disease (PUH ID); Shadyside Infectious Disease (SHY ID)

Potential donors were screened for the following transmissible agents in blood: fourth-generation combination HIV antigen and antibody immunoassay, human T-cell lymphotropic virus (HTLV) type 1 and 2, hepatitis A, hepatitis B, hepatitis C, cytomegalovirus, Epstein-Barr virus (EBV), syphilis, herpes simplex virus (HSV) type 1 and 2, human herpesvirus-6 (HHV-6), human polyomavirus-2 (JC virus), *Strongyloides stercoralis* and *Entamoeba histolytica*. The stool was screened for *Campylobacter, Escherichia coli* H7, *Salmonella, Shigella, Vibrio, Yersinia, Norovirus, Rotavirus, Clostridium difficile* toxin (with reflex PCR), *Helicobacter pylori, Adenovirus, Giardia, Cryptosporidia*, vancomycin-resistant enterococci (VRE), carbapenem-resistant enterobacteriacea (CRE) and extended-spectrum beta-lactamase (ESBL) bacterial species. PD-1 responder donors were additionally tested for nasal methicillin-resistant *Staphylococcus aureus* (MRSA). Pregnancy tests were conducted if the patient was female and not postmenopausal.

#### PD-1 responder donor stool sample processing and storage

PD-1 responder donor stool specimens were obtained every 14 days as illustrated in Fig. 1 **(Fig. 1: Donor and Recipient Screening Protocol).** A portion of each labeled specimen was used for infectious testing as outlined above and all stool specimens were processed in an anerobic hood. To create one FMT infusate, approximately 30g of donor stool was weighed on a calibrated digital scale. Sufficient sterile physiological saline (0.9% w/v of NaCl) and 85% glycerol were added for a final concentration of 10% of the fecal suspension. Fecal suspension was resuspended using a sterile spatula, passed through a 100μm cell strainer to remove particulate matter. The recovered fecal infusate was stored in a designated freezer at -80^0^C for up to 6 months. Prior to use, frozen fecal infusates were thawed for 20-30 minutes in a 25°C waterbath and transferred in a 50ml syringe with catheter tip. Unused samples beyond 6 months were discarded. Chain of custody logs that included details of all individuals who received and relinquished samples from processing till administration were maintained.

## Results

### Performance of screening algorithm and SARS-CoV-2 modification

Donor health was continuously monitored throughout their donation lifetime. FMT studies in nononcological indications have typically utilized bookend testing, wherein blood and stool tests are performed twice on either end of a collection period that typically spans 60 days. However, given the novel nature of this study (PD-1 refractory melanoma), the use of a previously uncharacterized source of FMT (PD-1 durable responders) and the uncertain performance of bookend testing in these circumstances, all tests were repeated every 14 days during the donor testing phase as illustrated in Fig. 1. Samples were quarantined until the donor passed screening at the end of the collection window.

**Fig. 2:**
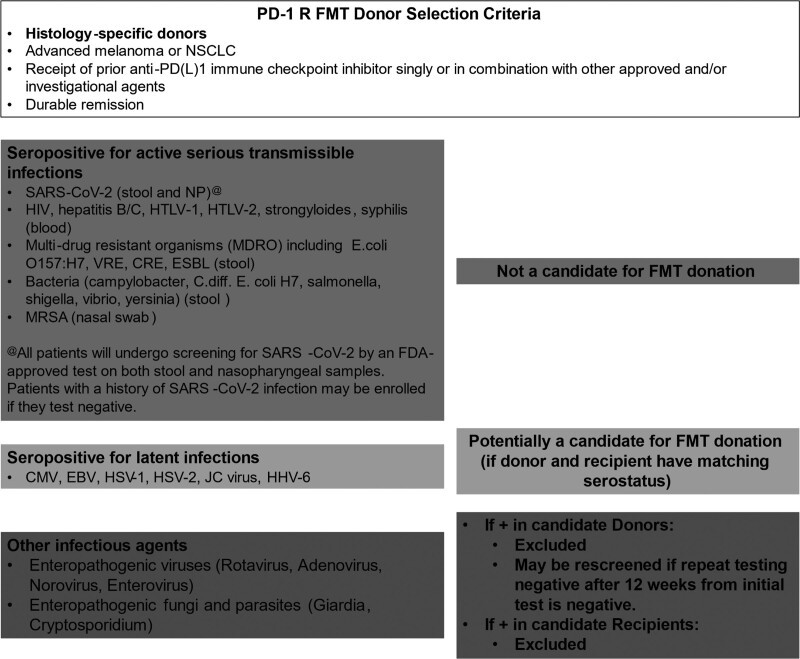
Donor and recipient matching protocol.

Donors who tested positive for serious blood-borne pathogens, enteric pathogens and multi-drug resistant organisms were permanently excluded from donation as illustrated in Fig. 2. Donors who screened positive for enteric pathogens (*Rotavirus, Adenovirus, Norovirus, Giardia, Cryptosporidium* and *Enterovirus*) were excluded regardless of symptoms but could be reconsidered following resolution of acute infection if they were asymptomatic, resumed normal stool patterns and repeat testing procedures were negative. Donors and recipients were sero-matched for latent viruses (cytomegalovirus, EBV, Hepatitis A/B/C, herpes simplex type 1/2, human herpesvirus 6, JC virus) as previously described [31]. Eligible PD-1 R donors provided repeat donations and underwent retesting every 2 weeks to ensure adequate sample availability in the stool bank (Fig. 1).

Following the outbreak of COVID-19, this clinical trial was initially halted before the development of a COVID-19/SARS-CoV-2 testing algorithm in discussion with the FDA and based on consensus guidelines [32–34] (Fig. 1). Given the uncertainties over potential SARS-CoV-2 fecal-oral transmission, the prior testing algorithm was altered to include additional screening questions to ascertain SARS-CoV-2 exposure in the donor/recipient questionnaire (questions 39–41, Supplementary Table 1, Supplemental digital content 1, http://links.lww.com/MR/A307), and patients were clinically assessed for COVID-19 by querying for fever, cough, dyspnea, chills, anosmia or ageusia, sore throat, muscle pain not otherwise explainable by alternative diagnosis within the previous 30 days per CDC recommendations [35]. Additionally, stool and nasopharyngeal specimens from both donors and recipients were tested using an FDA Emergency Use Authorization (EUA)-approved, Clinical Laboratory Improvement Amendments-certified COVID-19 diagnostic assay from the Diagnostic Solutions Laboratory. This reverse transcription PCR (RT-PCR) assay permitted qualitative detection of SARS-CoV-2 nucleic acids. Determination of ‘SARS-CoV-2 negative’ status was made on nasopharyngeal and stool specimens obtained at two timepoints: 1st sample was collected at least 14 days before stool sample donation, and the 2nd sample was collected at least 14 days after stool sample donation. The stool sample was released for FMT use no sooner than 48–72 h after 2nd test resulted and donors (and recipients) were deemed ‘SARS-CoV-2 negative’ (Fig. 1). Samples with discordant stool and nasopharyngeal SARS-CoV-2 results were excluded. Candidate donors (and recipients) who tested ‘SARS-CoV-2 positive’ were referred to infectious disease physicians for further treatment.

**Fig. 3:**
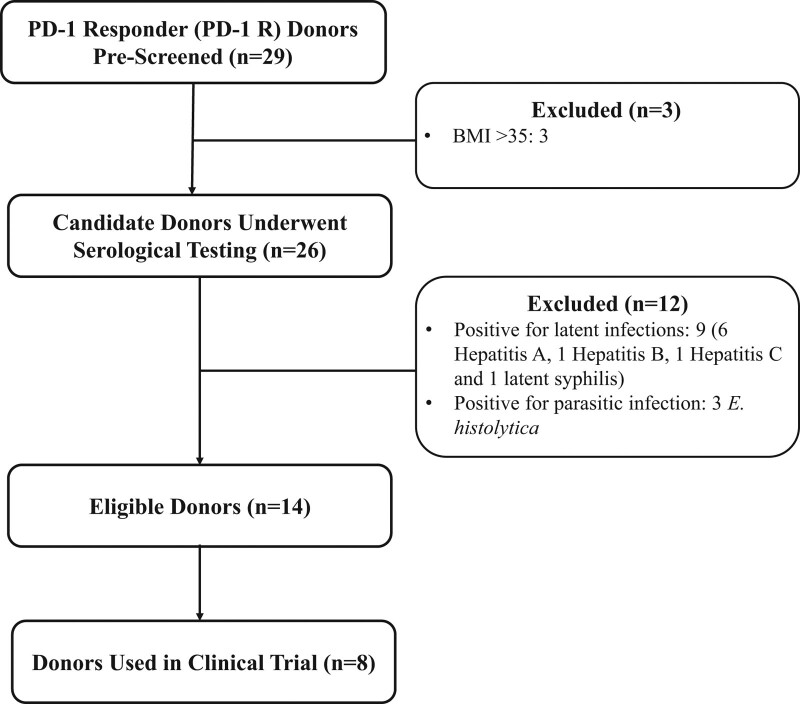
Consort Diagram Depicting Candidate Donors During Protocol.

Twenty-nine candidate PD-1 responder donors were evaluated, all of Caucasian ethnicity. Of these 29 donors, 26 passed prescreening and had a serological evaluation of blood and stool as summarized in Fig. 3. The three patients excluded in prescreening had BMI >35. Following initial serological studies, nine patients were excluded following the identification of potentially transmissible serious infections including hepatitis A (*n* = 6), hepatitis B, hepatitis C and syphilis (1 each) (Fig. 3). Each of these candidate donors was subsequently referred to infectious disease specialists for dedicated treatment and were successfully treated but not considered eligible as donors. Three patients with documented *E. histolytica* were successfully treated with antiparasitic agents but were also excluded as donors. Candidate PD-1 R donors who met all criteria and screened negative for serious infections including COVID-19/SARS-CoV-2 (in post-COVID period) were designated ‘PD-1 responder eligible donors’. Therefore, after the full screening of 29 candidate PD-1 responder donors, 14 PD-1 responder donors were identified as donors eligible to donate on a regular basis for the program for an acceptance rate of 48% (14/29).

### Results of infectious serologies on stool, blood and nasopharyngeal samples from donors and recipients

Among PD-1 responder donors, we observed the following seropositivity rates at pre- and post-COVID timepoints, respectively: cytomegalovirus (35.7 and 41.2%), EBV (96.4 and 81.5%), HSV-1 (32.1 and 37.0%), HSV-2 (14.3 and 3.7%), HHV-6 (100.0 and 92.6%) and JC virus (85.7 and 96.3%) (Table 2). Given that cancer patients are typically screened for exposure to chronic infectious hepatitis and HIV before exposure to anti-PD-1, seropositivity rates for Hepatitis B, C and HIV were unsurprisingly 0.0% at both pre- and post-COVID timepoints, respectively in both donors and recipients (Table 2). These results are similar to published seroprevalence statistics from healthy human blood donors in the USA – cytomegalovirus (45–100%) [36–38], EBV (80–100%) [39,40], HSV-2 (3–17%) [38,41–43] and HHV-6 (98–100%) [38,44]. However, the seroprevalence of HSV-1 in our cohort (32–37%) was lower than reported (67–82%) [38,41–43]. Conversely, the seroprevalence of the JC virus in our cohort (86–96%) was higher than reported (57–86%) [45,46]. Concordant with published seroprevalence statistics [47–49], seropositivity rates for HTLV-1, HTLV-2 and syphilis were very low at both pre- and post-COVID timepoints, respectively (Table 2).

**Table 2:** Results of infectious screening assays (blood)

Infectious Agent	Assay	Pre-COVID Donors			Pre-COVID Recipients			Post-COVID Donors			Post-COVID Recipients		
		Seropositive rate	Seronegative rate	*n*	Seropositive rate	Seronegative rate	*n*	Seropositive rate	Seronegative rate	*n*	Seropositive rate	Seronegative rate	*n*
Blood													
Cytomegalovirus (CMV)	CMV IgG	35.7%	64.3%	28	41.2%	58.8%	17	3.7%	96.3%	27	0.0%	100.0%	1
Epstein Barr virus (EBV)	EBV IgG and IgM	96.4%	3.6%	28	100.0%	0.0%	17	81.5%	18.5%	27	100.0%	0.0%	1
Entameba histolytica	E. histolytica IgG	3.6%	96.4%	28	0.0%	100.0%	17	22.2%	77.8%	27	0.0%	100.0%	1
Hepatitis A virus (HAV)	HAV total antibody (anti-HAV IgM and IgG)	0.0%	100.0%	28	23.5%	76.5%	17	11.1%	88.9%	27	100.0%	0.0%	1
Hepatitis B virus (HBV)	HBV surface antibody	3.6%	96.4%	28	11.8%	88.2%	17	0.0%	100.0%	27	0.0%	100.0%	1
Hepatitis B virus (HBV)	HBV surface antigen	0.0%	100.0%	28	0.0%	100%	17	0.0%	100.0%	27	0.0%	100.0%	1
Hepatitis B virus (HBV)	HBV core antibody	0.0%	100.0%	28	5.9%	94.1%	17	0.0%	100.0%	27	0.0%	100.0%	1
Hepatitis C virus (HCV)	Hepatitis C antibody	0.0%	100.0%	28	0.0%	100.0%	17	0.0%	100.0%	27	0.0%	100.0%	1
Herpes simplex virus (HSV) type 1 (HSV-1)	HSV-1 IgG	32.1%	67.9%	28	64.7%	35.3%	17	37.0%	63.0%	27	100.0%	0.0%	1
Herpes simplex virus (HSV) type 2 (HSV-2)	HSV-2 IgG	14.3%	85.7%	28	29.4%	70.6%	17	3.7%	96.3%	27	0.0%	100.0%	1
Human herpesvirus 6 (HHV-6)	HHV-6 IgG	100.0%	0.0%	28	76.5%	23.5%	17	92.6%	7.4%	27	100.0%	0.0%	1
Human polyomavirus 2 (JC virus)	JC virus IgG	85.7%	14.3%	28	88.2%	11.8%	17	96.3%	3.7%	27	100.0%	0.0%	1
Human T-lymphotropic virus (HTLV)-1	HTLV-1 IgG	0.0%	100.0%	28	0.0%	100.0%	17	0.0%	100.0%	27	0.0%	100.0%	1
Human T-lymphotropic virus (HTLV)-2	HTLV-2 IgG	0.0%	100.0%	28	0%	100.0%	17	0.0%	100.0%	27	0.0%	100.0%	1
HIV	5th generation IgG with reflex ELISA	0.0%	100.0%	28	0%	100.0%	17	0.0%	100.0%	27	0.0%	100.0%	1
Strongyloides stercoralis	S. stercoralis IgG	0.0%	100.0%	28	0%	100.0%	17	0.0%	100.0%	27	0.0%	100.0%	1
Syphilis	RPR, IgM, IgG	0.0%	100.0%	28	0%	100.0%	17	0.0%	100.0%	27	0.0%	100.0%	1

We extensively evaluated donors and recipients for multiple enteric pathogens including *S. stercoralis* and *E. histolytica* in blood; and *Campylobacter, E. coli* O157:H7, *Salmonella, Shigella, Vibrio, Yersinia, Norovirus, Rotavirus, C. difficile, H. pylori, adenovirus, Giardia* and *Cryptosporidia.* We noted a 3.6% incidence of *E. histolytica*, 3.6% incidence of norovirus, 3.7% incidence of giardia and 7.1% incidence of *C. difficile* in donors across pre- and post-COVID timepoints (Tables 2 and 3). These particular samples were discarded, the patients referred for specific therapy where appropriate, and upon re-screening, the patients were found to be negative although these donors were excluded. No recipients tested positive for any enteric pathogens. We also evaluated both donors and recipients for MDROs (CRE, VRE, ESBL) in stool and MRSA in nasopharyngeal samples. The incidence of MDROs including MRSA among donors and recipients was 0.0%, lower than reported point prevalence rates of MRSA colonization among hospital-exposed patients in an urban, nonoutbreak setting [50,51].

**Table 3:** Results of infectious screening assays (stool)

Infectious agent	Assay	Pre-COVID Donors			Pre-COVID Recipients			Post-COVID Donors			Post-COVID Recipients		
		Seropositive rate	Seronegative rate	n	Seropositive rate	Seronegative rate	n	Seropositive rate	Seronegative rate	n	Seropositive rate	Seronegative rate	n
Stool													
Campylobacter species	GI pathogen panel	0%	100%	28	0%	100%	17	0%	100%	27	0%	100%	1
Clostridium difficile (C. diff)	C. diff toxin with reflex PCR	7.1%	92.9%	28	0%	100%	17	0%	100%	27	0%	100%	1
Escherichia coli O157:H7	E. coli O157:H7	0%	100%	28	0%	100%	17	0%	100%	27	0%	100%	1
Helicobacter pylori (H. pylori)	H. pylori antigen with reflex ELISA	0%	100%	28	0%	100%	17	0%	100%	27	0%	100%	1
Salmonella	GI pathogen panel	0%	100%	28	0%	100%	17	0%	100%	27	0%	100%	1
Shigella	GI pathogen panel	0%	100%	28	0%	100%	17	0%	100%	27	0%	100%	1
Vibrio	GI pathogen panel	0%	100%	28	0%	100%	17	0%	100%	27	0%	100%	1
Yersinia	GI pathogen panel	0%	100%	28	0%	100%	17	0%	100%	27	0%	100%	1
vRotavirus	GI pathogen panel	0%	100%	28	5.9%	94.1%	17	0%	100%	27	0%	100%	1
Adenovirus	Adenovirus DNA PCR	0%	100%	28	0%	100%	17	0%	100%	27	0%	100%	1
Norovirus	GI pathogen panel	3.6%	96.4%	28	0%	100%	17	0%	100%	27	0%	100%	1
Giardia	Ova and parasite screen	0%	100%	28	0%	100%	17	3.7%	96.3%	27	0%	100%	1
Cryptosporidia	Ova and parasite screen	0%	100%	28	0%	100%	17	0%	100%	27	0%	100%	1
Vancomycin-resistant Enterococcus (VRE)	VRE screen	0%	100%	28	0%	100%	17	0%	100%	27	0%	100%	1
Carbapenem-resistant Enterobacteriaceae (CRE)	CRE screen	0%	100%	28	5.9%	94.1%	17	0%	100%	27	0%	100%	1
Extended-spectrum β-lactamase-producing Enterobacteriaceae (ESBL)	ESBL screen	0%	100%	28	5.9%	94.1%	17	0%	100%	27	0%	100%	1
SARS-CoV-2	COVID-19 rtPCR	N/A	N/A	N/A	N/A	N/A	N/A	11.1%	88.9%	27	0%	100%	1

Following the COVID-19 epidemic and reports of excretion and viability of SARS-CoV-2 in feces and association with infection [52,53], we modified the donor/recipient questionnaire to include additional screening questions to evaluate for SARS-CoV-2 exposure (questions 39–41, Supplementary Table 1, Supplemental digital content 1, http://links.lww.com/MR/A307), and additionally screened for SARS-CoV-2 both by nasopharyngeal swab and in stool using an FDA EUA-approved RT-PCR assay. The incidence of SARS-CoV-2 in stool was 11.1% in donors (3 of 27 specimens) and 0.0% in recipients; and the incidence in nasopharyngeal samples was 0.0% in donors and 0.0% in recipients (Tables 3 and 4). Of note, the three donors who tested positive for SARS-CoV-2 did so only on the stool and not nasopharyngeal swabs. All three donors had screened negative on the screening questionnaire, were afebrile, otherwise asymptomatic and had no SARS-CoV-2-specific recent exposures. Per CDC guidance, these samples were discarded and patients were advised to self-quarantine. All three donors were rescreened 12 weeks later and were negative for SARS-CoV-2 in both stool and nasopharyngeal specimens. The 11.1% positivity rate among candidate stool donors for an FMT program has not previously been reported.

**Table 4:** Results of infectious screening assays (nasopharynx)

Infectious agent	Assay	Pre-COVID Donors			Pre-COVID Recipients			Post-COVID Donors			Post-COVID Recipients		
		Seropositive rate	Seronegative rate	*n*	Seropositive rate	Seronegative rate	*n*	Seropositive rate	Seronegative rate	n	Seropositive rate	Seronegative rate	n
Nasal													
MRSA		0%	100%	28	0%	100%	17	0%	100%	27	0%	100%	1
SARS-CoV-2	COVID-19 RT-PCR	N/A	N/A	N/A	N/A	N/A	N/A	0%	100%	27	0%	100%	1

### Cost of infectious testing

The cost of a full infectious work-up at our center was approximately US $2260.24 per donor per timepoint not including SARS-CoV-2 testing, and US $2460.24 per donor per timepoint if SARS-CoV-2 testing was included (Table 5). The total costs of screening all donors and 16 recipients were $129 713.20 and $40 884.32, respectively; and the cost of identifying a single eligible donor was $9 265.23.

**Table 5:** Cost of donor and/or recipient screening

Infectious agent	Assay	CPT codes	Cost (per patient per time point)
Blood			
Cytomegalovirus (CMV)	CMV IgG	86644	25.00
Epstein Barr virus (EBV)	EBV IgG and IgM	866650000, 866650000	27.86
Entameba histolytica	E. histolytica IgG	867530000	143.00
Hepatitis A virus (HAV)	HAV total antibody (anti-HAV IgM and IgG)	86708 & 86709	42.00
Hepatitis B virus (HBV)	HBV surface antibody	86706	18.00
Hepatitis B virus (HBV)	HBV surface antigen	87340	18.00
Hepatitis B virus (HBV)	HBV core antibody	86704	21.00
Hepatitis C virus (HCV)	Hepatitis C antibody	86803	25.00
Herpes simplex virus (HSV) type 1 (HSV-1)	HSV-1 IgG	86694 and 86695	31.95
Herpes simplex virus (HSV) type 2 (HSV-2)	HSV-2 IgG	86696	34.00
Human herpesvirus 6 (HHV-6)	HHV-6 IgG	875320000	511.00
Human polyomavirus 2 (JC virus)	JC virus IgG	877980000	46.00
Human T-lymphotropic virus (HTLV)-1	HTLV-1 IgG	86687	15.00
Human T-lymphotropic virus (HTLV)-2	HTLV-2 IgG	86688	25.00
Human immunodeficiency virus (HIV)	5th generation IgG with reflex ELISA	86703 & 87390	55.00
Strongyloides stercoralis	S. stercoralis IgG	866820000	10.67
Syphilis	RPR, IgM, IgG	867800000	8.69
Stool			
Campylobacter species	GI pathogen panel	870460000	2.34
Clostridium difficile (C. diff)	C. diff toxin with reflex PCR	87324	20.00
Escherichia coli O157:H7	E. coli O157:H7	870460000	1.48
Helicobacter pylori (H. pylori)	H. pylori antigen with reflex ELISA	87338	24.00
Salmonella	GI pathogen panel	870450000	5.23
Shigella	GI pathogen panel	870450000	5.23
Vibrio	GI pathogen panel	87046	2.34
Yersinia	GI pathogen panel	870460000	1.40
Rotavirus	GI pathogen panel	86759	23.00
Adenovirus	Adenovirus DNA PCR	877980000	350.00
Norovirus	GI pathogen panel	877990000	736.00
Giardia	Ova and parasite screen	87329	21.00
Cryptosporidia	Ova and parasite screen	87328	21.00
Vancomycin-resistant Enterococcus (VRE)	VRE screen	870810000	6.00 (bundled with CRE, ESBL)
Carbapenem-resistant Enterobacteriaceae (CRE)	CRE screen	870810000	
Extended-spectrum β-lactamase-producing Enterobacteriaceae (ESBL)	ESBL screen	870810000	
SARS-CoV-2	SARS-CoV-2	86769	100.00
Nasopharynx			
Methicillin-resistant Staphylococcus aureus	MRSA screening culture	87641	62.00
SARS-CoV-2	SARS-CoV-2	86769	100.00
Total Cost (Pre-COVID, all donors)			63 286.72
Total Cost (Post-COVID, all donors)			66 426.48
Total Cost (Pre-COVID, all recipients)			38 424.08
Total Cost (Post-COVID, all recipients)			2460.24
Total Cost (Pre-COVID, per donor/recipient)			2260.24
Total Cost (Post-COVID, per donor/recipient)			2460.24

## Discussion

The link between gut dysbiosis and both immune-related adverse events and nonresponse to cancer immunotherapy across multiple settings including anti-PD-1 ICI and anti-CD19 chimeric antigen receptor T-cell therapy is increasingly appreciated [10,54,55]. This has spurred multiple clinical trials aimed at evaluating various microbiome therapeutics including healthy donor or responder-derived FMT; complete consortia products (SER-401, NCT03817125; MaaT033, NCT03772899); synthetic bacterial consortia (VE800, NCT04208958) and monoclonal microbials (CBM588, NCT03829111 and NCT05122546; EDP1503, NCT03595683; MRx0518, NCT03775850). Given the early data demonstrating the efficacy of responder FMT in treating PD-1 nonresponsive melanoma, there is great interest in studying this in other oncologic indications and multiple FMT trials are ongoing in various settings including mismatch repair deficient (dMMR) tumors following progression on anti-PD-1 ICI (NCT04729322), gastrointestinal cancers following progression on anti-PD-1 ICI (NCT04130763) and treatment-naïve metastatic melanoma patients in combination with anti-PD-1 ICI (NCT03772899). Hence, there is an increasing demand to establish and maintain FMT donor pools at individual cancer centers.

Of the initial 29 donors who were screened, following the exclusion of three for excessive BMI, and 12 for infectious issues, 14 eligible donors were identified, eight of whom were utilized in the clinical trial. The overall efficiency of screening was 48% (14/29) and higher than what has been reported with FMT programs to treat refractory *C. difficile* infection at single centers (10–37%) [23,24,26], and considerably higher than the <3% reported by OpenBiome, the longest standing international stool bank [56]. The observed screening efficiency, which was not significantly impacted by the COVID-19 pandemic, suggests that a well-resourced, organized, screening program based out of an academic institution should be able to generate sufficient responder-derived donor material for clinical trial purposes.

In the context of healthy donor FMT programs established to treat rCDI, the cost of screening 1 donor has been reported as ranging from US $440 to US $885 [24,25]. Herein, we report that the cost of screening a single donor was US $2460.24 per donor per timepoint or $9265.23 to identify a single eligible donor. The greater costs reported herein are likely related to the extensive infectious serological studies (Table 1). Overall, this argues that the cost of developing a responder-derived donor fecal bank is considerable and far greater than similar approaches using healthy donor material, particularly if commercially sourced from stool banks such as OpenBiome.

In hematological malignancy patients following allogeneic hematopoietic cell transplant (allo-HCT), FMT post-allo-HCT is associated with the expansion of donor-derived taxa and restoration of gut microbiota diversity [57,58]. Separately, FMT has been successfully investigated as a treatment for steroid-refractory acute GI graft-versus-host disease as well [59,60]. However, even in this immunocompromised population with paramount concerns for infectious complications, the incidence of FMT-related bacteremia is low [57]. Separately, no instances of FMT-related infectious complications were reported in either of the two immuno-oncologic studies that evaluated FMT administration to advanced cancer patients along with anti-PD-1 in refractory melanoma [19,20]. Given the low observed prevalence of certain GI pathogens (such as *Campylobacter, H. pylori, Salmonella, Shigella, Yersinia, Adenovirus, Cryptosporidia*, etc.), it may be preferable to use a bookend testing procedure (i.e. testing before and after multiple stool donations) with an appropriate window (i.e. 60 days) while quarantining stool samples until the post-donation tests are confirmed negative.

We observed an 11.1% positivity rate of asymptomatic SARS-CoV-2 in the stool of candidate FMT donors. Given that SARS-CoV-2 can be found in feces [61], the potential risk of fecal transmissibility [62], and that these patients denied significant exposures and screened negative for concerning symptoms, the reported positivity rate strongly argues for repeated screening of donor stools through a molecular test for SARS-CoV-2 on both stool and nasopharyngeal specimens at each donation period with a relatively short window such as 14 days. It is important for the FMT stool banks, FMT clinical team, treating physicians and FMT recipients to recognize that a negative test for a pathogen does not fully mitigate the risk of transmission via donated material. Concurrently, it is equally important for FMT stool banks and FMT clinical teams to periodically update their laboratory testing as more sensitive and/orspecific tests are developed and as new infectious agents are deemed transmissible via the fecal route (e.g. monkeypox virus) [63].

Limitations of this study include the small sample size and the reasons for exclusion which may not necessarily be generalizable to other nononcology patient populations. The screening costs were accurate as this is a single-center study and the information pertaining to codes was readily available, but may not be applicable to non-US locations.

There is growing interest in developing microbiome-based therapeutic approaches in immuno-oncology to treat ICI resistance. Unlike the rCDI setting where the use of healthy donor FMT from central stool banks with storage and shipment of frozen samples is cost-effective and feasible [64], the data in the immuno-oncologic setting has been generated primarily with ICI responders and not healthy donors. Hence the development of an ICI responder-derived FMT program must consider the factors implicit in the care of a cancer patient including the need for longitudinal follow-up and the oncologist-patient relationship; suggesting that such programs may be better suited to being locally situated rather than centrally organized. These data suggest that the creation and maintenance of an ICI responder-derived FMT program are feasible. Full transparency and reporting of screening protocols are critical to enable clinicians and regulators to determine the acceptability and efficacy of such stool banks and further development of ICI responder-derived FMT programs.

## Acknowledgements

The authors thank the patients and their caregivers who participated in this study, as well as the research teams at the UPMC Hillman Cancer Center. The research presented herein was funded in part through the Melanoma Research Foundation Breakthrough Consortium Award (D.D.), Jack Buncher Foundation (D.D.), NCI ETCTN Pittsburgh Cancer Consortium (PCC) Support Grant UM1 CA186690 (D.D.), NIH/NCI U01 CA268806 (H.M.Z. and D.D.) and R01 CA222203 (H.M.Z).

IRB approval number CR19060111-007, Hillman Cancer Center protocol number 17-034.

D.F.: data curation, formal analysis, writing–original draft. M.P.A.: data curation, formal analysis. D.H., M.S., H.D., C.H., S.B., S E., and A.R.: data curation. H.Z.: investigation, resources, data curation. D.D.: supervision, investigation, resources, data curation, formal analysis, writing–original draft, review and editing.

### Conflicts of interest

D.D.: research support: Arcus, Bristol-Myers Squibb, Checkmate Pharmaceuticals, CellSight Technologies, Merck, GlaxoSmithKline/Tesaro, Zucero Therapeutics; Scientific Advisory Board: Vedanta Biosciences; consultancy with compensation: Checkmate Pharmaceuticals, Finch Inc., Immunocore, Shionogi and Vedanta Biosciences; Patents: (both provisional) U.S. Patent Application No. 63/208,719 (Compositions and Methods for Determining Responsiveness to Immune Checkpoint Inhibitors (ICI), Increasing Effectiveness of ICI, and Treating Cancer), and Application No. 63/124,231 (Compositions and Methods For Treating Cancer). H.Z.: Bristol-Myers Squibb, Checkmate Pharmaceuticals, and GlaxoSmithKline (research support) and Bristol-Myers Squibb, Checkmate Pharmaceuticals, GlaxoSmithKline, and Vedanta (consulting).

For the remaining authors, there are no conflicts of interest.

## Supplementary Material


