# Supplementary material for: Screening costs associated with donor selection for fecal microbiota transplantation for treatment of PD-1 refractory melanoma patients
Source: Melanoma Res. 2023 Feb 17;33(2):136–48. doi: 10.1097/CMR.0000000000000871 (PMC9949524; doi:10.1097/CMR.0000000000000871)
Supplement: Supplementary file 1 [file mr-33-136-s001.pdf]

Supplementary Table 1: FMT Recipient (or Donor) Screening Questionnaire

Dear *** :

You have been identified as a potential donor for a medical procedure called fecal microbiota transplantation (FMT, or stool transplantation). FMT is a relatively new medical procedure that has been used to treat patients with C. difficile infections (CDI) – a diarrheal illness caused by antibiotics that kill the normal stool bacteria of the human gastrointestinal tract. The composition of gut bacteria has been shown to mediate outcomes to PD-1 inhibitor therapy in animal models of melanoma. You have been identified by Dr. Diwakar Davar and Dr. Hassane Zarour as a potential stool donor.

Your doctors are conducting a study in which patients with advanced melanoma whose cancer **is not responding** to PD-1 therapy receive FMT. In this study, selected patients will receive an extract from your frozen stool sample. Are you interested in learning more about how you can participate as a donor for FMT?
YES, PLEASE / NO THANKS

If no, you do not need to answer any more questions. Please return this form to the person who gave it to you or to the return address at the end of this form. If yes, please continue to answer the following questions:

**Name (please print)**	
**Date of birth (MM/DD/YYYY format):**	
**Gender (please circle):**	**Male**<br>**Female**
**Telephone numbers (please state):**	
**Date (MM/DD/YYYY format) and time completed:**	

The answers to the following questions will be used by our stool transplant physician to determine if your feces can be used for a stool transplant. The answers to some but not all questions may disqualify you as a donor. Please answer the questions truthfully. The answers that you give will NOT be shared with the person you are donating to or appear in his/her chart.

1. Are you at least 18 years old? **YES/NO**
2. Except for birth control, do you take any prescription medications? **YES/NO**
3. What is your height in feet and inches?

4. What is your weight in pounds?

5. Have you ever donated blood before?
   a. If yes, have you even been notified by the blood bank of any positive testing for infectious conditions such as hepatitis C or HIV?
   b. If no, have you attempted to donate blood previously but asked not to donate for medical reasons ?

Please answer the following **YES / NO** questions to the best of your ability:

In the past 6 weeks

6. Female donors: Have you been pregnant or are you pregnant now? **YES / NO / I AM MALE**

In the past 8 weeks have you:

7. Donated blood, platelets or plasma? **YES / NO**
8. Had any vaccinations or other shots? **YES / NO**
9. Had contact with someone who had a smallpox vaccination? **YES / NO**

In the past 3 months have you:

10. Used any antibiotics? **YES / NO**
11. Had any diarrhea? **YES / NO**
12. Had any vomiting? **YES / NO**
13. Had any food poisoning? **YES / NO**
14. Had any fevers? **YES / NO**
15. Been hospitalized (long enough to stay overnight)? **YES / NO**
16. Been seen in an emergency room or urgent care visit? **YES / NO**

In the past 12 months have you

17. Had a blood transfusion? **YES / NO**
18. Had a transplant such as organ, tissue, or bone marrow? **YES / NO**
19. Had a graft such as bone or skin? **YES / NO**
20. Come into contact with someone else's blood? **YES / NO**
21. Had an accidental needle-stick? **YES / NO**
22. Had sexual contact with anyone who has HIV/AIDS or has had a positive test for the HIV/AIDS virus? **YES / NO**
23. Had sexual contact with a prostitute or anyone else who takes money or drugs or other payment for sex? **YES / NO**
24. Had sexual contact with anyone who has ever used needles to take drugs or steroids, or anything not prescribed by their doctor? **YES / NO**
25. Had sexual contact with anyone who has hemophilia or has used clotting factor concentrates? **YES / NO**
26. Had sexual contact with a person who has hepatitis? **YES / NO**
27. Lived with a person who has hepatitis? **YES / NO**
28. Had a tattoo? **YES / NO**
29. Had ear or body piercing? **YES / NO**
30. Had or been treated for syphilis or gonorrhea? **YES / NO**
31. Been in juvenile detention, lockup, jail, or prison for more than 72 hours? **YES / NO**

In the past three years have you

32. Been outside the United States or Canada? **YES / NO**

From 1980 through 1996,

33. Did you spend time that adds up to three (3) months or more in the United Kingdom? (Northern Ireland, Wales, Scotland, England [sometimes called Great Britain])? **YES / NO**
34. Were you a member of the U.S. military, a civilian military employee, or a dependent of a member of the U.S. Military? **YES / NO**

From 1980 to the present, did you

35. Spend time that adds up to five (5) years or more in Europe? **YES / NO**
36. Receive a blood transfusion in the United Kingdom or France? **YES / NO**

From 1977 to the present, have you:

37. Received money, drugs, or other payment for sex? **YES / NO**
38. Male donors: had sexual contact with another male, even once? **YES / NO / I AM FEMALE**

COVID-19 Related Exposure

39. Have you at any time been diagnosed with laboratory-confirmed SARS-CoV-2 infection?
40. Have you at any time experienced symptoms of COVID-19 (e.g., fever, cough, shortness of breath) not explained adequately by another diagnosis?
41. Have you been exposed to a suspected or confirmed case of COVID-19 or SARS-CoV-2 infection?

Have you EVER:

42. Had *C. difficile* infection previously? **YES / NO**
43. Had a positive test for the HIV/AIDS virus? **YES / NO**
44. Used needles to take drugs, steroids, or anything not prescribed by your doctor? **YES / NO**
45. Used clotting factor concentrates? **YES / NO**
46. Had hepatitis? **YES / NO**
47. Had malaria? **YES / NO**
48. Had Chagas' disease? **YES / NO**
49. Had babesiosis? **YES / NO**
50. Received a dura mater (or brain covering) graft? **YES / NO**
51. Had any type of cancer, including leukemia? **YES / NO**
52. Had parents, brothers, sisters, or children diagnosed with cancer? **YES / NO**
53. Had any problems with your heart or lungs? **YES / NO**
54. Had a bleeding condition or a blood disease? **YES / NO**
55. Had sexual contact with anyone who was born in or lived in Africa? **YES / NO**
56. Been in Africa? **YES / NO**
57. Been in the Caribbean? **YES / NO**
58. Been in Central or South America (anywhere south of the US Border including Mexico)? **YES / NO**
59. Been elsewhere outside the US? **YES / NO**
60. Had relatives who had Creutzfeldt-Jakob disease? **YES / NO**
61. Been previously tested for HIV or viral hepatitis? **YES / NO**
62. Tested positive for MRSA (methicillin resistant *Staphylococcus aureus*), VRE (vancomycin resistant enterococcus) or other resistant bacteria? **YES / NO**
63. Had diarrhea lasting for longer than 2 weeks? **YES / NO**
64. Had irritable bowel syndrome? **YES / NO**
65. Had chronic constipation? **YES / NO**
66. Had chronic pain? **YES / NO**

A physician will review this form to determine if you can be scheduled for a screening visit with an infectious disease physician for additional questions. If you answer in the positive to questions 39-41, you will be referred to an infectious disease physician for additional evaluation. If you are eligible based on this visit, you will be scheduled for laboratory testing. If you are eligible and willing to participate as a potential stool donor, you will be asked to do the following:

1) Schedule a screening visit with a physician in Oakland at the Falk Medical Building
2) Have blood tests and stool tests collected for testing
3) Donate stool either in person or off-site on the morning of the scheduled procedure.

Please mail this form to:

ATTN: Diwakar Davar, MD
UPCI Fecal Transplant Program

Hillman Cancer Center, UPCI Research Pavilion
5117 Center Avenue
Suite 1.32d
